# Bronchiectasis exacerbation study on azithromycin and amoxycillin-clavulanate for respiratory exacerbations in children (BEST-2): study protocol for a randomized controlled trial

**DOI:** 10.1186/1745-6215-14-53

**Published:** 2013-02-20

**Authors:** Anne B Chang, Keith Grimwood, Andrew C Wilson, Peter P van Asperen, Catherine A Byrnes, Kerry-Ann F O’Grady, Theo P Sloots, Colin F Robertson, Paul J Torzillo, Gabrielle B McCallum, Ian B Masters, Helen M Buntain, Ian M Mackay, Jacobus Ungerer, Joanne Tuppin, Peter S Morris

**Affiliations:** 1 Child Health Division, Menzies School of Health Research, Charles Darwin University, Darwin, NT, Australia; 2 Queensland Children’s Respiratory Centre, Royal Children’s Hospital, Brisbane, QLD, Australia; 3 Queensland Children’s Medical Research Institute, Brisbane, QLD, Australia; 4 Queensland Paediatric Infectious Diseases Laboratory, Royal Children’s Hospital, Brisbane, QLD, Australia; 5 Department of Respiratory Medicine, Princess Margaret Hospital, Perth, Australia; 6 Department of Respiratory Medicine, The Children’s Hospital at Westmead and Sydney Medical School, University of Sydney, Sydney, NSW, Australia; 7 Department of Paediatrics, University of Auckland and Starship Children’s Hospital, Auckland, New Zealand; 8 Department of Respiratory Medicine, Royal Children’s Hospital, Murdoch Children’s Research Institute, University of Melbourne, Melbourne, VIC, Australia; 9 Royal Prince Alfred Hospital, Sydney, Australia; 10 Department Chemical Pathology, Queensland Pathology, Royal Brisbane Hospital, Brisbane, Australia; 11 Department of Paediatrics, Royal Darwin Hospital, Darwin, NT, Australia

**Keywords:** Amoxycillin-clavulanate, Azithromycin, Bronchiectasis, Placebo, Pulmonary exacerbations, Randomized controlled trial

## Abstract

**Background:**

Bronchiectasis unrelated to cystic fibrosis (CF) is being increasingly recognized in children and adults globally, both in resource-poor and in affluent countries. However, high-quality evidence to inform management is scarce. Oral amoxycillin-clavulanate is often the first antibiotic chosen for non-severe respiratory exacerbations, because of the antibiotic-susceptibility patterns detected in the respiratory pathogens commonly associated with bronchiectasis. Azithromycin has a prolonged half-life, and with its unique anti-bacterial, immunomodulatory, and anti-inflammatory properties, presents an attractive alternative. Our proposed study will test the hypothesis that oral azithromycin is non-inferior (within a 20% margin) to amoxycillin-clavulanate at achieving resolution of non-severe respiratory exacerbations by day 21 of treatment in children with non-CF bronchiectasis.

**Methods:**

This will be a multicenter, randomized, double-blind, double-dummy, placebo-controlled, parallel group trial involving six Australian and New Zealand centers. In total, 170 eligible children will be stratified by site and bronchiectasis etiology, and randomized (allocation concealed) to receive: 1) azithromycin (5 mg/kg daily) with placebo amoxycillin-clavulanate or 2) amoxycillin-clavulanate (22.5 mg/kg twice daily) with placebo azithromycin for 21 days as treatment for non-severe respiratory exacerbations. Clinical data and a parent-proxy cough-specific quality of life (PC-QOL) score will be obtained at baseline, at the start and resolution of exacerbations, and on day 21. In most children, blood and deep-nasal swabs will also be collected at the same time points. The primary outcome is the proportion of children whose exacerbations have resolved at day 21. The main secondary outcome is the PC-QOL score. Other outcomes are: time to next exacerbation; requirement for hospitalization; duration of exacerbation, and spirometry data. Descriptive viral and bacteriological data from nasal samples and blood inflammatory markers will be reported where available.

**Discussion:**

Currently, there are no published randomized controlled trials (RCT) to underpin effective, evidence-based management of acute respiratory exacerbations in children with non-CF bronchiectasis. To help address this information gap, we are conducting two RCTs. The first (bronchiectasis exacerbation study; BEST-1) evaluates the efficacy of azithromycin and amoxycillin-clavulanate compared with placebo, and the second RCT (BEST-2), described here, is designed to determine if azithromycin is non-inferior to amoxycillin-clavulanate in achieving symptom resolution by day 21 of treatment in children with acute respiratory exacerbations.

**Trial registration:**

Australia and New Zealand Clinical Trials Register (ANZCTR) number http://ACTRN12612000010897. http://www.anzctr.org.au/trial_view.aspx?id=347879

## Background

Bronchiectasis unrelated to cystic fibrosis (CF) is an heterogenous condition with many initiating events. It is now considered more common than thought previously, especially in developing countries [1], in socioeconomically disadvantaged Indigenous communities in affluent countries [2] and as a co-morbidity of other chronic pulmonary diseases, such as asthma [3,4] and chronic obstructive pulmonary disease (COPD) [5]. Bronchiectasis affects all age groups, including infants [6]. Although the consequences of bronchiectasis are predominantly related to respiratory morbidity, there are also independent cardiovascular effects, such as coronary artery disease [7,8], which are likely to worsen with the systemic inflammation arising from chronic pulmonary infection.

Over the past 15 to 20 years, it has been increasingly recognized that non-CF bronchiectasis is a major contributor to chronic respiratory morbidity [9,10] and mortality [11,12] worldwide [2,13]. In our recently completed multicenter study of 346 Australian children newly referred for chronic cough and managed using a standardized protocol [14], 31 (9%) had radiologically proven bronchiectasis [15]. In the USA, the average number of bronchiectasis-associated hospitalizations increased by 2 to 3% per year between 1993 and 2006, and during this period, the average age-adjusted annual hospitalization rate was 16.5 per 100,000 of the population [9]. In some populations from affluent countries, the prevalence of bronchiectasis is one of the highest reported in the world, such as in Central Australia, where 1 in 68 Indigenous children are affected [16]. Similarly, in New Zealand, the estimated national prevalence rates of bronchiectasis are much higher in Maori (1 in 2300) and Pacific Island (1 in 625) children than in New Zealand European (1 in 7440) children aged under 15 years [17]. The minimum national incidence of non-CF bronchiectasis before the age of 15 years is 1 in 1,700 births, compared with 1 in 3,179 births for a diagnosis of CF in New Zealand [17]. In the European Union and the USA, the prevalence of CF is 7.4 to 7.9 per 100,000 (whole population data) [18]. Indeed, there are far more patients with bronchiectasis than patients with CF attending respiratory services globally [19]. In the US, about 30,000 [20] people have CF, whereas over 110,000 people have non-CF bronchiectasis [13]. Moreover, the latter is likely to be an underestimate, as many cases of non-CF bronchiectasis are misdiagnosed. or co-exist with asthma [3,4,18] and COPD [5].

Effective clinical management reduces both short-term and long-term morbidity (and probably mortality) associated with bronchiectasis [1,21-23]. Cohort data have shown that about 80% of newly diagnosed adults (non-smokers) with bronchiectasis reported symptoms dating back to childhood, and that the duration of chronic cough (the most common symptom of bronchiectasis [24]) was related (r = −0.51, *P*<0.001 in non-smokers) to lung function at diagnosis [25]. Arguably, appropriate overall management and treatment of exacerbations (leading to a reduction in persistent symptoms) potentially prevents or reduces deterioration of chronic respiratory disease [26]. Our study and a London-based retrospective study both found that with appropriate treatment in specialized centers, lung function improves and can be maintained [22,23]. However, despite substantial improvements, those with poor lung function at diagnosis were still likely to have poor lung function 5 years later [23]. We also found that the only significant predictor of decline in forced expiratory volume in 1 second (FEV_1_) was the frequency of hospitalized exacerbations, and that FEV_1_ percentage predicted declined by 1.95% with each hospitalized exacerbation [23]. In addition to the biologic effects of respiratory exacerbations, these episodes also impair quality of life (QOL) and well-being. as shown by deteriorating scores for QOL and on the Depression, Anxiety and Stress Scale (DASS) during exacerbations [27]. Taken together, as airway injury in children is superimposed upon the physiological changes involving lung growth and development [28,29], we speculate that early and effective management of bronchiectasis exacerbations in children may lead to reduced hospitalizations, better QOL, and improved future adult lung function.

Although most respiratory physicians will treat acute exacerbations intensively with antibiotics and airway clearance [24,30], some exacerbations are caused by viral infections, and may not require antibiotic therapy. However, it is possible that viral-bacterial interactions in the airways could promote or prolong endobronchial bacterial infection, which, with the accompanying inflammatory cascade, is a risk for additional lung injury [26,31]. To address whether antibiotics are superior to placebo at providing short-term clinical benefits, the first component of our Bronchiectasis Exacerbation Study (BEST), is a multicenter randomized controlled trial (RCT; BEST-1) designed to answer the question: ‘Does azithromycin or amoxycillin-clavulanate, compared with placebo, improve the resolution of respiratory exacerbations on day 14 of treatment?’ [32].

The second component of BEST (BEST-2) seeks to determine whether the two antibiotics used in BEST-1 are equivalent at achieving resolution of an acute respiratory exacerbation in children with bronchiectasis, compared with placebo. Based on available, but limited, data on the microbiology of lower-airway secretions in children with non-CF bronchiectasis [33], amoxycillin-clavulanate is currently recommended as the first-line empirical oral-antibiotic treatment for non-severe bronchiectasis exacerbations in children [24]. However, amoxycillin-clavulanate requires dosing two to three times per day, and causes gastrointestinal symptoms in many children. Oral azithromycin is attractive as an alternative first-line therapy because of its long half-life, markedly reduced dosing schedule, and good safety profile in children [34]. Although azithromycin can be used 3 days/week [35] or even once weekly [36], our proposed study uses a daily dose in order to maintain effective blinding of the medications. However, if daily azithromycin is shown to be equivalent to amoxicillin-clavulanate, then extended-interval azithromycin dosage schedules could be trialed in appropriate clinical settings where reduced dosing frequency is particularly appealing, such as for patients likely to have poor adherence.

As well as having anti-bacterial activity against most respiratory bacterial pathogens associated with non-CF bronchiectasis [37], azithromycin has additional anti-microbial properties that could prove beneficial. These include its unique bactericidal activity against intracellular strains of non-typeable *Haemophilus influenzae*[38], inhibitory effects upon biofilm formation [39], and anti-viral properties [40]. Furthermore, as a member of the macrolide class of antibiotics, it also has immunomodulatory and anti-inflammatory functions [41]. Given the emerging importance of both airway inflammation and biofilm development in the pathogenesis of acute and chronic respiratory disease [26,42], azithromycin may be a valuable intervention. Nevertheless, no RCTs have yet been carried out for azithromycin to help determine its role in treating acute exacerbations of bronchiectasis. Despite the lack of high-level evidence, azithromycin is sometimes used to treat adults with bronchiectasis exacerbations, perhaps as an extension of its reported positive effects on acute exacerbations of COPD [43]. By contrast, two RCTs of maintenance azithromycin therapy have been conducted in adults with non-CF bronchiectasis. One was a small, non-blinded, double cross-over RCT, where 6 months of azithromycin therapy was shown to improve lung function and reduce exacerbation frequency [44]. More recently, a 6-month, parallel, double-blind, placebo-controlled trial in New Zealand adults with bronchiectasis reported that azithromycin decreased exacerbation events, but did not alter lung function or QOL measures [45].

### Aims of the study

In the second phase of BEST (BEST-2) the primary question will be: ‘Is daily oral azithromycin non-inferior (within a 20% margin) to oral amoxycillin-clavulanate at achieving resolution of exacerbations by day 21 of treatment?’

The secondary aims are similar to those in BEST-1 [32], and are to: 1) determine the effect of azithromycin or amoxycillin-clavulanate on QOL, systemic inflammation, time to next respiratory exacerbation, and duration of exacerbations; 2) examine factors that predict response to the two antibiotics, including respiratory pathogens (viruses, bacteria, macrolide-resistant bacteria) present in respiratory secretions, and systemic markers of inflammation; and 3) describe, by using sensitive molecular detection techniques, the point prevalence and diversity of respiratory viruses and *Mycoplasma pneumoniae* and *Chlamydiales* species during exacerbations, compared with the findings at enrolment when the children are clinically stable.

The study will test the primary hypothesis that oral azithromycin is non-inferior (within a 20% margin) to oral amoxycillin-clavulanate at achieving resolution of respiratory exacerbations by day 21 of treatment in children with non-CF bronchiectasis.

## Methods

### Study design

**Figure 1:**
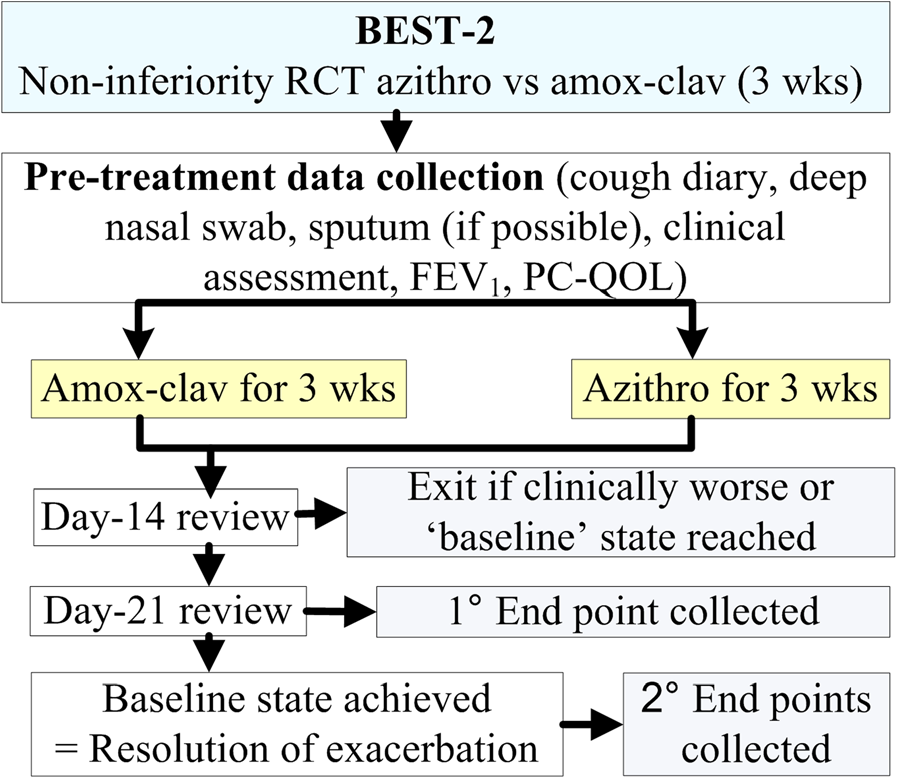
**Overall schematic study design.** Amox-clav, amoxycillin-clavulanate; Azithro, azithromycin; BEST, Bronchiectasis Exacerbation Study.

We are conducting a multicenter, parallel group, double-dummy, double-blind placebo RCT (with concealed allocation) to assess whether oral azithromycin is non-inferior to oral amoxycillin-clavulanate at treating children with a non-severe exacerbation of bronchiectasis. Our study plan is summarized in Figure 1.

### Eligibility

The inclusion criteria are: age less than 19 years at time of enrolment; diagnosis of bronchiectasis by a respiratory physician following high-resolution computed tomography in the 5 years immediately prior to study entry, or if diagnosed earlier, evidence of regular follow-up by a respiratory physician for treatment of bronchiectasis; and more than two respiratory exacerbations in the 18 months prior to study entry. Children who have participated in BEST-1 [32] may participate subsequently in BEST-2. These children will be re-randomized for BEST-2.

Exclusion criteria are: current or recent severe exacerbation of bronchiectasis (dyspnea, hemoglobin desaturation <90% in air or hospitalization) in the 8 weeks immediately prior to study entry; presence of CF or liver dysfunction; hypersensitivity to beta-lactam or macrolide antibiotics; current or recent (in the 4-months before study enrolment) lower-airway infection by a member of the *Pseudomonas* genus of gram-negative bacteria; receipt of beta-lactam or macrolide antibiotics within the 3 weeks preceding study entry; or current treatment for cancer.

### Recruitment

Eligible children will be identified from clinics at one of six sites (Brisbane, Darwin, Melbourne Perth and Sydney in Australia, and Auckland in New Zealand). Parents will be approached, and informed consent obtained. Baseline pre-exacerbation data will be collected (Figure 1), parents will be contacted monthly, and children will be reviewed every 3 months. Parents will be educated specifically on how to recognize the symptoms of an acute respiratory exacerbations, and asked to contact the research nurse at the onset of an exacerbation event.

### Intervention and follow-up

A double-dummy design is planned. If eligibility is fulfilled, and after informed consent has been obtained, the child will be randomized to one of two arms. At the start of the exacerbation, the child will receive orally: 1) azithromycin with placebo amoxycillin-clavulanate or 2) amoxycillin-clavulanate with placebo azithromycin. The azithromycin dose is 5 mg/kg/day (to a maximum of 200 mg daily, and for amoxycillin-clavulanate it is 22.5 mg/kg/dose twice daily (maximum 900 mg/dose). Equivalent volumes of placebo will be given for both trial medications. All treatments will continue for 21 days.

An exacerbation is defined as an increase in sputum volume or purulence, or change in cough (>20% increase in cough score [46] or type (dry to wet) [47]) for more than 3 days. We validated this definition in our prospective study, and found that the kappa values (between clinicians) of these symptoms and signs were excellent (>0.75) [48]. Daily diaries will also be collected during exacerbations until the scores for 2 or more days reflect the child’s baseline state, which for each child will be established at enrolment, prior to any exacerbations. This assessment consists of a combination of symptoms (daily cough (yes/no), cough quality (wet/dry/none), cough score [46] averaged over two consecutive days) and signs (sputum color (if any present) using a color chart card (BronkoTest Ltd, London, UK), and crackles on chest auscultation). Children will be reviewed on days 14 and 21, and at resolution of the exacerbation. The exacerbation is considered resolved when symptoms and signs are the same as the baseline state. Post-exacerbation, the children will be followed up and evaluated clinically every 3 months for a period of 18 months or until their next exacerbation (whichever is sooner). The time to next exacerbation will be determined by duration of days from the resolution of the current exacerbation to the beginning of the next exacerbation.

### Randomization, allocation, and blinding

Upon enrolment, the child will be assigned to the next unique number on the appropriate stratified list. The allocation will be performed by the trial pharmacist at the Royal Children’s Hospital (Brisbane, Australia). Randomization is stratified by site (Brisbane, Darwin, Melbourne, Perth, Sydney, Auckland), age (≤5 or >5 years) and underlying etiology (idiopathic/post-pneumonia or all other causes). The randomization sequence is computer-generated and uses permuted blocks. The allocation sequence will be concealed at all times throughout the study. The computer-generated allocation sequence was prepared by a statistician external to the study team.

The specially manufactured placebo medications (Institute of Drug Technology Australia Ltd, Melbourne, Victoria, Australia), have a similar taste and color to their respective antibiotics. Both active medications (azithromycin and amoxycillin-clavulanate) will be repackaged and relabeled so that both antibiotics and their respective placebos are provided in identical opaque bottles. For all trial medications, equal volumes of water are added using a syringe and needle by punching the seal. Adherence will be assessed by parent report and return of empty bottles.

### Data collection

All data will be recorded on standardized forms. On enrolment, demographic information (including age, gender, ethnicity, and household size), birth history, breast-feeding history, prior illness, and *in utero* and household smoke exposure, will be recorded, and a physical examination will be performed by a study physician. The primary and secondary outcome measures will be collected at the time points specified above. Serious and non-serious adverse effects (AEs: nausea, vomiting, diarrhea, rash) will also be documented and monitored. Safety exit points are discussed under ‘End points’ below.

### Specimen collection

At enrolment (baseline) all children will have a deep-nasal swab (NS) specimen collected. In a subset, additional specimens will be collected at baseline and during exacerbations, depending upon feasibility (some children may be unable to attend the study center at the onset of the exacerbation) and willingness of parents to allow additional NS collections and venepuncture procedures to be performed. These specimens are:

A deep NS specimen for respiratory viruses, respiratory bacterial pathogens (including antibiotic susceptibility testing), and other potentially important respiratory pathogens (*M. pneumoniae, Chlamydiales* spp) at baseline, and at the beginning and resolution of an exacerbation. The techniques used are identical to previous studies [49-51], in which the specimens were described as nasopharyngeal swabs. In this study, we have elected to call these specimens ‘deep-nasal swabs’ as it is anatomically accurate to do so. The NS specimens will be handled in accordance with our research laboratory protocol (see below).

Blood samples will be taken at baseline and at the beginning and end of each exacerbation for determination of C-reactive protein (CRP), interleukin (IL)-6 (a neutrophilic marker of inflammation [52]), serum amyloid A (SAA) [48], and markers of viral infection (IL-10, interferon γ-inducible protein (IP)-10) [53].

Sputum samples will also be taken at baseline and at the beginning and end of each exacerbation (when possible) for lower-airway microbiology cultures and antibiotic sensitivity tests.

### Further description of scores and laboratory methods

#### Cough score

The verbal categorical descriptive score is a validated daily diary score of cough rated on a six-point scale (0 (no cough) to 5 (severe cough and cannot perform usual activities)) with increasing scores reflecting greater interference with usual activities. This rating was validated against an objective cough-meter measure [46], and changes in cough scores have been shown to reflect changes in objective cough counts [54].

#### Parent-proxy cough-specific quality of life score

The parent-proxy cough-specific quality of life (PC-QOL) score is a 27-item questionnaire designed to assess the level of frequency of feelings (15 items) and worry (12 items) related to their child’s cough. It uses a seven-point Likert-type scale, with higher scores reflecting less frequency and fewer worry concerns (that is, greater QOL) [55,56]. The minimal important difference is 0.62 as determined by the distribution method, and 0.9 as determined by the anchor method [57].

#### Bacteriology of nasal swab

Compared with NS, oropharyngeal sampling underestimates *Streptococcus pneumoniae* carriage by approximately 50% [58], Thus NS is the preferred method when evaluating the presence of antibiotic-resistant bacteria. Culturing, identifying, and when appropriate, serotyping common respiratory bacteria are established techniques in our research laboratory [51,59]. Swabs are stored in skim-milk tryptone-glucose-glycerol broth medium at −80°C ,before being batch-processed for typical respiratory bacterial pathogens, notably *S. pneumoniae*, *H. influenzae (*including strains of non-typeable *H. influenzae)* and *Moraxella catarrhalis.* Batches of swabs are thawed, and 10 μL aliquots cultured overnight on selective media at 37°C in 5% CO_2_. Growth of *S. pneumoniae*, *H. influenzae* and *M. catarrhalis* is recorded and confirmed by standard techniques [51,60]. Two isolates each of *S. pneumoniaH. influenzae* and *M. catarrhalis* per positive swab are tested for anti-microbial resistance and stored [51,60]. *S. pneumoniae* isolates are serotyped using the Quellung method (antisera from Statens Serum Institute, Copenhagen, Denmark). Routine susceptibility testing using the calibrated dichotomous susceptibility disk-diffusion method. If the if the azithromycin disk annulus is less than 6 mm the minimum inhibitory concentration (MIC) of azithromycin will also be determined (Etests; AB Biodisk, Solna, Sweden). For *S. pneumoniae*, the penicillin MIC will be determined for penicillin non-susceptible isolates (oxacillin and/or penicillin disk annulus < 6 mm) and for *H. influenzae*, the ampicillin MIC will be determined for ampicillin non-susceptible isolates (ampicillin disk annulus < 6mm). Interpretive criteria (Clinical and Laboratory Standards Institute breakpoints) used for *S. pneumoniae* are penicillin non-susceptible MIC greater 0.12 μg/ml and azithromycin resistance MIC 2 μg/ml or greater; and for *H. influenzae*, ampicillin resistance MIC 4 μg/ml or greater and azithromycin resistance MIC greater than 4 μg/ml. A nitrocephin-based test will identify beta-lactamase activity in *H. influenzae* and *M. catarrhalis* isolates.

#### Assessment for viruses and other bacteria

We will use our previously described methods of assessment [61,62]. Nucleic acids will be extracted from the media (High Pure Viral Nucleic Acid Kit; Roche Diagnostics, Sydney, NSW, Australia), in accordance with the manufacturer’s instructions. Real-time PCR assays will be used to detect respiratory syncytial viruses (A and B), adenoviruses, influenza viruses (A and B), parainfluenza, human metapneumovirus, human coronaviruses (OC43, HK1, 229E, and NL63), enteroviruses, rhinoviruses (including determining specific rhinovirus genotypes by sequencing the VP4-VP2 region [63]) and the more recently described human viruses (human bocavirus 1, parechoviruses, and human polyomaviruses K1 and WU) and *M. pneumoniae* and *Chlamydiales* species [64].

#### Blood markers

CRP, threshold 5 mg/l) are standard tests that will be analyzed by the Diagnostic Laboratories of each participating center. SAA, IL-6 (threshold <3 pg/ml), IL-10 (threshold <0.5 pg/ml) and IP-10, (threshold 2.8 pg/ml) will be performed by commercial enzyme immunoassay kits (R&D Systems, Minneapolis, USA) at our research laboratory.

Spirometry (in children aged ≥5 years) will be performed using American Thoracic Society criteria and the recorded FEV_1_ % predicted. We elected not to use oscillatory measures, as we previously found no difference in airway resistance between steady and exacerbation states [48]. Thus, we will use conventional spirometry, although we do not expect to detect significant differences.

### End points

Participation will be complete when the child’s clinical state returns to baseline and the time to next exacerbation has been obtained. Other exit points are: if the child deteriorates during treatment prior to day 21, or becomes sufficiently intolerant of the trial medications to require withdrawal from the study (as determined by the treating clinician).

### Outcome measures

#### Primary outcome

The primary outcome will be the proportion of children whose exacerbations have resolved by day 21 of treatment. Exacerbations will be considered resolved when symptoms and signs are the same as the baseline state. Children who are withdrawn from the study, or receive additional antibiotic treatment, will be categorized as non-resolved.

#### Secondary clinical outcomes

The main secondary outcome is the PC-QOL score. Other outcomes are 1) the time to next exacerbation; 2) requirement for hospitalization; 3) duration of exacerbation (persistence of symptoms till return to baseline state); and 4) FEV_1_ % predicted.

#### Secondary laboratory outcomes

Serum markers (CRP, SAA, IL-6, IL-10, IP-10) and data on viruses and respiratory bacterial pathogens, including their antibiotic susceptibility to penicillin and azithromycin.

### Sample size

We plan to enroll 170 children (85 per arm), providing 90% power (α = 0.05, 1-sided) with 20% non-inferiority margin to detect 80% resolution rate by day 21. The margin selected is relatively large in statistical terms, but the physicians considered it clinically appropriate. As the primary outcome will be obtained in all enrolled children, retention fraction has not been factored in for the intention-to-treat analysis.

The main secondary outcome (secondary aim 1) is PC-QOL. Based on a non-inferiority limit of 0.9 (minimum important difference [57]) and standard deviation of 0.9, our sample size provides a power of 99.9% (α = 0.05, one-sided 95% CI) for data from at least 136 children (assuming at least 80% retention of children enrolled). For secondary aim 2 (see list under ‘Aims of the study’), we will be examining eight main factors, and thus a sample size of 136 exceeds the recommended minimum (n = 10 per factor) [65]. The eight factors are: smoking, age, underlying etiology, detection of virus (any versus none, then single versus multiple viruses), presence of azithromycin resistance, and levels of various blood markers (IL-6, IL-10, IP-10).

### Statistical analysis and reporting

Data will be reported and presented in accordance with the updated Consolidated Standards of Reporting Trials (CONSORT) criteria [66]. Children will be analyzed in accordance with allocation status (regardless of subsequent management).

For our primary aim, the main effects of the interventions will be determined by comparing the primary outcome (resolution of exacerbation) between groups (azithromycin versus amoxicillin-clavulanate). Children who exit the study as ‘clinically worse’ or ‘drop-out’ before the end point is reached will be considered as ‘non-resolved’. Those who exit the study as ‘returned to baseline’ will be considered as ‘resolved’. Odds ratios will be calculated and, if appropriate, the number needed to treat (for benefit) will be expressed. Per-protocol analysis will be an *a priori* secondary analysis.

#### Statistical analysis for secondary outcomes and aims

For the clinical secondary outcome (secondary aim 1), the *t*-test or the Mann–Whitney test will be used for continuous variables (depending on normality of data distribution). A Kaplan-Meier curve will be constructed for each group for time to resolution and time to next exacerbation, as reported previously [67]. For secondary aim 2 (factors that predict response to antibiotics), univariate analyses will be used to examine several biologic factors (for example, smoking, age, ethnicity, underlying etiology, detection of virus (any versus none, then single versus multiple viruses), presence of azithromycin resistance, and levels of blood markers (IL-6, IL-10, IP-10)). Factors with *P*<0.2 will be included in a logistic regression model. Potential interactions (for example,. between viruses and bacteria) will be examined in the model. Descriptive data will be used for secondary aim 3 (point prevalence of respiratory pathogens).

### Data safety monitoring committee

A Data Safety Monitoring Committee has been established and met prior to commencement of this study.

### Ethics approval

The protocol has received ethics approval from the respective Human Research Ethics Committees of all the participating institutions (Brisbane: Children’s Health Queensland Hospital and Health Service (Royal Children’s Hospital) and University of Queensland; Darwin: Department of Health and Families and Menzies School of Health Research; Melbourne: Royal Children’s Hospital; Perth: Princess Margaret Hospital; Sydney: Sydney Children’s Hospital Network Human Research Ethics Committee; and Auckland: Northern Ethics Committee, Ministry of Health and Starship Children’s Health local ethics committee). The study is being conducted under Australia’s Therapeutic Goods Administration Clinical Trial Notification (CTN) scheme.

## Discussion

In addition to the increased recognition over the past 20 years of the disease burden from bronchiectasis, the need for more effective treatment is also reflected by 1) the association of the disease with other chronic pulmonary disorders; 2) its adverse effect on QOL and other co-morbidities; and 3) its influence on lung-function decline and mortality [68].

However, despite the considerable global burden of bronchiectasis and the importance of exacerbations [68], there are no published RCTs on the management of bronchiectasis exacerbations in children [69]. Bronchiectasis is often considered a neglected disease, and services to manage people with bronchiectasis receive disproportionately fewer allocated resources (both clinical and research), compared with other chronic pulmonary disorders [10,70,71]. Almost all current recommendations are based on management of CF [24,30], and such extrapolation can, on occasions, be detrimental for those with non-CF bronchiectasis. For example, a large RCT found that deoxyribonuclease (efficacious for CF) increased exacerbations and accelerated the decline in FEV_1_ in adults with bronchiectasis [72], despite previous case reports advocating its use [73].

In asthma [61], CF [74] and COPD [75,76], viral triggers of acute exacerbations are well described; however, no such data exist for non-CF bronchiectasis. Whether other potential respiratory pathogens (*M. pneumoniae* and *Chlamydiales* species) trigger exacerbations is also unknown. Our retrospective study found that 34% of exacerbations were preceded by a viral-like illness [77]. Thus, for the first time in this population, BEST-1 [32] and BEST-2 will both determine the nature and diversity of respiratory viruses and *M. pneumoniae and Chlamydia species* associated with bronchiectasis exacerbations, and compare these results with those obtained at baseline, to help determine attributable risk.

The 20% margin used for our study, although relatively large, was deemed clinically appropriate, given the potential major advantage of future once-weekly dosing with azithromycin compared with twice-daily dosing wit amoxycillin-clavulanate. In disadvantaged communities, adult-supervised twice-daily dosing is not always possible, and may result in children with exacerbations receiving suboptimal treatment. By contrast, once-weekly dosing is feasible with weekly home or clinic visits; we have achieved this outcome in studies involving remote Indigenous settings in Australia [50] and in New Zealand [78]. Thus, if non-inferiority between amoxycillin-clavulanate and azithromycin is established, this would provide an easier option for managing exacerbations, and could substantially improve adherence in disadvantaged settings.

### Rationale for our chosen outcome measures and timeframe

In BEST-1, we chose day 14 as the time point for this RCT, based on available data from our retrospective data of 115 respiratory exacerbations [77], and on parental and healthcare professional concerns over using placebo for an extended period [32]. For BEST-2, presented here, we chose day 21 as the crucial time point because hospitalization is usually recommended if there is no symptomatic improvement after 3 to 5 weeks of oral-antibiotic therapy.

Adult bronchiectasis studies show that QOL measures, particularly cough-specific QOL indices, are valid and important outcome measures [79,80]. Likewise, we have previously shown the utility of the PC-QOL score in children with bronchiectasis [27].

## Conclusion

Our study addresses a large knowledge gap in an under-researched area [71]. Our multi-center, double-blind, double-dummy, RCT, which examines the non-inferiority of azithromycin compared with amoxycillin-clavulanate for acute non-severe exacerbations of bronchiectasis in children has the potential to simplify the management of these exacerbations, thus possibly achieving both short-term gains and a long-term benefit for reducing the morbidity of bronchiectasis. Conclusive results will strengthen evidence-based, standard treatment guidelines.

### Trial status

Recruitment for BEST-1 [32] started in mid-March 2012 in Darwin, and in June 2012 in Brisbane. Randomization for the BEST-2 component commenced in October 2012 in Brisbane and Darwin.

## Abbreviations

BEST: Bronchiectasis Exacerbation Study; CF: Cystic fibrosis; COPD: Chronic obstructive pulmonary disease; CRP: C-reactive protein; FEV_1_: Forced expiratory volume in 1 second; IL: Interleukin; IP: Interferon γ-inducible protein; MIC: Minimum inhibitory concentration; NS: Nasal swab; PC-QOL: Parent-proxy cough-specific quality of life; PCR: Polymerase chain reaction; QOL: Quality of life; RCT: Randomized controlled trial; SAA: Serum amyloid A.

## Competing interests

The authors declare that they have no financial competing interests related to this study.

## Authors’ contributions

AC conceived the study, participated in its design and coordination, and drafted the manuscript. PM, CR, KG, PvA, AW, KO, PT, TS participated in study design and submission to the National Health and Medical Research Council (NHMRC). GM participated in initiating the project, and TS and IMM participated in the viral analysis plan. IBM, CB, and HB will assist in recruitment and assessment of the children. JU will participate in the biochemical analysis of the blood samples. All authors have read and approved the final manuscript.
